# Prognostic Factors and Outcome Measures After Rituximab Therapy in Central Nervous System Vasculitis: A Systematic Review

**DOI:** 10.7759/cureus.69936

**Published:** 2024-09-22

**Authors:** Amal M Alharthi, Ziad Aljundi, Fatimah A Alharbi, Khadija E Alfaqih

**Affiliations:** 1 Neurology, King Abdullah Medical City, Mecca, SAU

**Keywords:** anti-neutrophil cytoplasmic antibody, cns vasculitis, polyangiitis, primary angiitis of the central nervous system (pacns), rituximab

## Abstract

Given the growing popularity of Rituximab (Rmab) treatment as a potential substitute for cyclophosphamide, we conducted this review to determine aspects related to Rmab therapy in central nervous system vasculitis (CNSV) patients, aiming to establish both the beneficial and detrimental consequences of Rmab while providing clinical guidance for managing patients' conditions.

This systematic review was prepared following the Preferred Reporting Items for Systematic Reviews and Meta-Analyses (PRISMA) guidelines. The PubMed, Web of Science, and Scopus databases were utilized to investigate Rmab treatment in CNSV from January 2015 to May 2024. The research question was structured using the Population, Intervention, Comparison, Outcome, and Study Design (PICOS) criteria. Case series with only three or more unique cases, prospective or retrospective non-randomized studies, and randomized controlled trials (RCTs) were addressed.

The initial evaluations were performed in PubMed, Scopus, and Web of Science. After removing duplicate entries, 177 publications were obtained, and 41 were chosen for full-text assessment. The review then incorporated information gathered from 27 studies, including 4 case series, 15 non-randomized cohorts, and 8 RCTs. Rmab is generally regarded as effective for CNSV therapy. Given its success, induction Rmab therapy is now frequently employed as maintenance therapy for CNSV patients.

Rmab is a viable option for the induction of remission and maintenance treatment, with a successful reduction in relapse rates.

## Introduction and background

Central nervous system vasculitis (CNSV) in adulthood is an uncommon idiopathic inflammatory condition that exclusively affects intracranial and spinal cord vessels, mostly tiny and medium-sized. Clinical symptoms include focal neurological impairments, headache, symptomatic epilepsy, and cognitive impairment, and the diagnosis is typically confirmed by biopsy or angiogram. The pathological forms include lymphocytic, granulomatous, and necrotizing. The relatively low prevalence and diagnostic challenges have resulted in a scarcity of prospective randomized trials on CNSV in adults [1].

The underlying cause of CNSV remains mostly unclear. Infectious pathogens, such as varicella-zoster, have been linked to CNSV. Nevertheless, the specific pathway of infection that causes CNSV has not been determined. Primary vasculitis of the central nervous system (PVCNS) is recognized as a noninfectious condition that differs from infectious or secondary CNSV. Histological investigations have shown that various immune cells infiltrate cerebral arteries, leading to unique histopathologic subgroups. For example, the necrotizing vasculitis variant is usually linked to vessel wall damage and intracerebral hemorrhage [2].

The clinical characteristics used to diagnose CNSV include headache, impaired cognition, and localized neurological impairments [2,3]. The diagnostic criteria proposed by Calabrese and Mallek involve several requirements. First, there must be acquired, unexplained neurological or psychiatric deficits. Second, classic angiographic or histopathological features of CNS vasculitis must be present. Lastly, there should be no evidence of systemic vasculitis or other conditions that could contribute to or mimic the angiographic or histopathological features of CNS vasculitis [4]. The PVCNS diagnosis can be confirmed with a biopsy or an angiogram. The latest version of diagnostic standards highlights the use of CSF in determining the PVCNS diagnosis by revealing signs of CNS inflammation, such as elevated WBC count and protein levels, while also excluding other potential causes like infections or systemic vasculitis [3].

Anti-neutrophil cytoplasmic antibody (ANCA)-associated vasculitis (AAV) is an autoimmune illness that causes inflammation in small to medium-sized blood vessels. Granulomatosis with polyangiitis (GPA), also referred to as Wegener's granulomatosis, microscopic polyangiitis (MPA), and eosinophilic GPA (EGPA), commonly known as Churg-Strauss syndrome, are characterized by a lack of tolerance to neutrophil granule proteins like PR3 or MPO [5]. In Europe, 46 to 184 people are diagnosed with AAV annually, with incidence rates ranging from 2.1 to 14.4 per million for GPA, 2.4 to 10.1 per million for MPA, and 0.5 to 3.7 per million for EGPA [6]. A five-year survival rate of 74%-91% for GPA, 45%-76% for MPA, and 60%-97% for EGPA is anticipated [7]. Current induction techniques achieve remission in 70-90% of patients but can cause adverse reactions, particularly in older, lung-impaired individuals [8]. Maintenance treatment reduces relapses, although half of the patients experience a recurrence within five years [9]. High-dose glucocorticoids (GC) and cyclophosphamide (CYC) or Rituximab (Rmab) are the standard therapeutic options for new-onset AAV [10]. Non-life-threatening illnesses can be treated with methotrexate (MTX) and mycophenolate mofetil (MMF), which, while promoting remission, are more likely to induce relapse than CYC or Rmab [11].

Rmab is a monoclonal antibody that can suppress CD20+ B lymphocytes for more than 24 weeks. Following FDA approval in 2011, Rmab offered a new approach for treating AAV patients. It was approved at a dose of 375 mg/m^2^/week for four weeks, along with GC [12]. Given the growing popularity of Rmab treatment as a potential substitute for CYC, we conducted this review to determine aspects related to Rmab treatment in CNSV patients, establishing both beneficial and detrimental consequences of Rmab while providing clinical guidance for managing patients' conditions.

## Review

Study protocol

This systematic review was prepared following the Preferred Reporting Items for Systematic Reviews and Meta-Analyses (PRISMA) [13].

Focused question

The research question was structured using the Population, Intervention, Comparison, Outcome, Study Design (PICOS) criteria: "What are the prognostic factors and outcomes of Rmab therapy in managing CNS vasculitis?"

Information sources and search strategy

The PubMed, Web of Science, and Scopus databases were utilized for any studies related to Rmab therapy in CNS vasculitis from January 2015 to May 2024, using the following combination of Medical Subject Headings (MeSH) phrases: ("Primary CNS Vasculitis," OR "Anti-Neutrophil Cytoplasmic Antibody-Associated Vasculitis," OR "ANCA-associated Vasculitis," OR "Microscopic Polyangiitis," OR "Polyangiitis" OR "Wegener's Granulomatosis" OR "Granulomatosis with Polyangiitis" OR "PR3-AAV" OR "MPO-AAV") AND ("Anti-CD20," OR "Rituximab"). Case series with only three or more unique cases were addressed. Each publication’s citations were manually reviewed to find studies not found in web-based databases. Two independent reviewers conducted the evidence-based investigation and accomplished the screening procedure in compliance with pre-established requirements.

Eligibility criteria

The eligibility criteria employed were determined according to the PICOS criteria. The population considered included patients with CNS vasculitis or AAV. The intervention evaluated was a treatment protocol consisting of Rmab for the management of CNSV or AAV. The comparison involved examining the prognostic factors and outcomes with various treatment protocols for CNSV or AAV. The outcome focused on Rmab prognostic, safety, and efficacy studies in CNSV and AAV.

Study design

Case series (3+ cases), prospective or retrospective non-randomized studies, and randomized controlled trials (RCTs) were included. Systematic reviews, questionnaires, pilot studies, case reports, commentaries, and animal models were excluded. Non-English articles, research that did not meet our eligibility requirements, and studies published before 2015 were also excluded.

Study selection

The two authors independently evaluated all paper titles and abstracts and selected full-text papers. Non-qualifying publications were deleted. Also, pertinent published studies were searched in the references of the reviewed studies. Reviewers resolved conflicts. In case of dispute, a third reviewer was sought to decide. Each of the three evaluators agreed on their final decision. A quantitative analysis (meta-analysis) was not feasible due to the methodologies of the studies.

Data extraction and synthesis

The first author's name, study title, journal name, publication year, country of study, study design, treatment regimen, remission or relapses, other measures of outcome, and study inference were collected using a standardized data collection protocol. Two independent investigators extracted data independently to minimize errors. The corresponding authors were contacted for additional details.

Results

**Figure 1:**
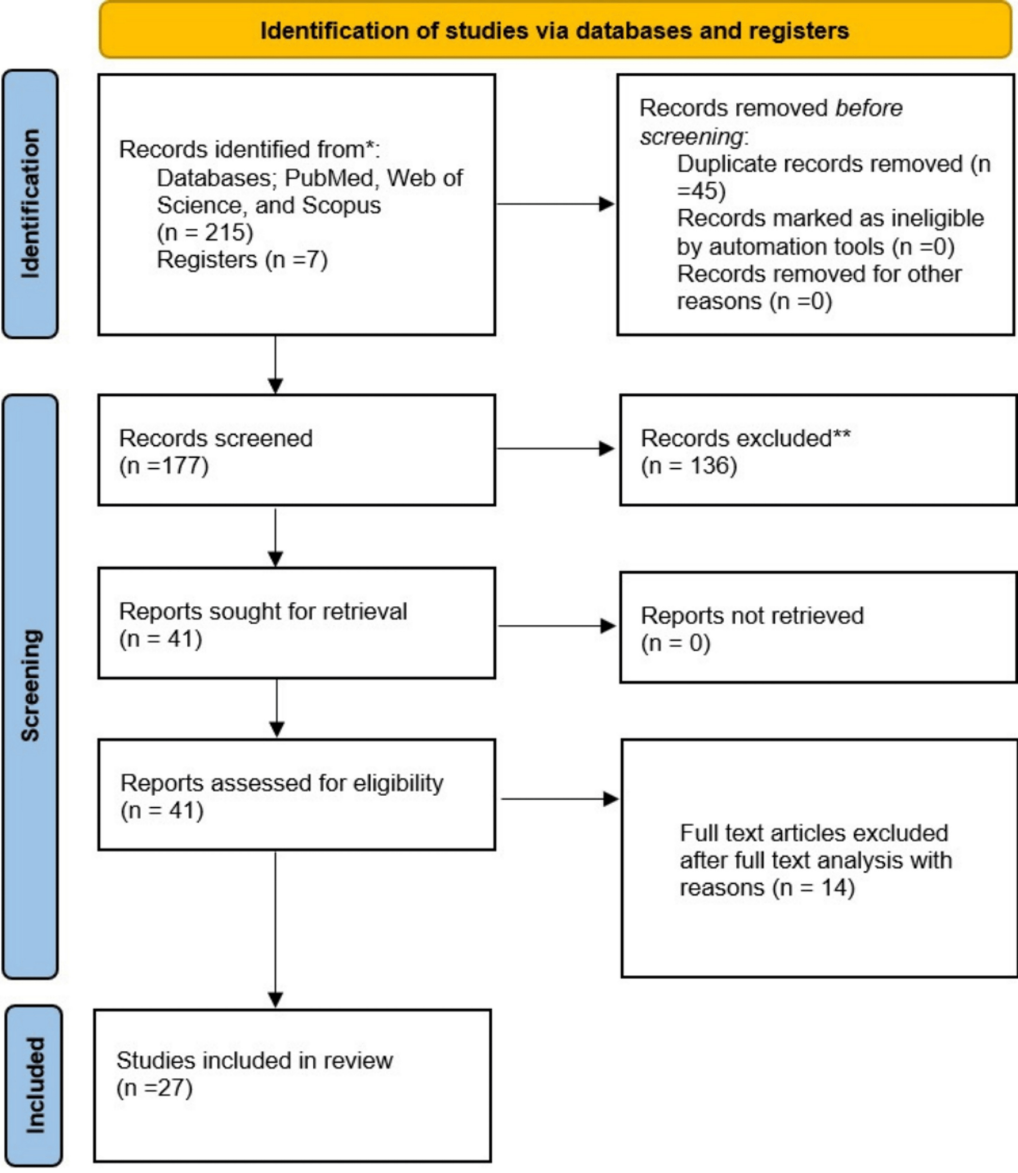
PRISMA flow chart of the included studies. PRISMA: Preferred Reporting Items for Systematic Reviews and Meta-Analyses.

The initial evaluations were performed in PubMed, Scopus, and Web of Science. After removing duplicate entries, 177 publications were obtained, 136 were eliminated based on titles and abstracts, and 41 were chosen for full-text assessment. The analysis then excluded review articles (n=9), overlapping syndromes with other autoimmune disorders (n=3), and those missing full texts (n=2). The review then contained the information gathered from 27 studies [14-40]. Figure 1 depicts the PRISMA-compliant selection procedure. The study includes four case series [14-17], 15 non-randomized cohorts [18-32], and eight RCTs [33-40].

Tables 1-2 summarize case series and non-randomized studies on the prognostic and outcome measures of Rmab in CNS vasculitis and AAV patients. Table 3 lists all reviewed RCTs in CNS vasculitis and AAV patients and contextualizes relapses and remissions. Rmab is usually regarded as effective for CNSV and AAV therapy. Given its success, induction Rmab therapy is now frequently employed as a maintenance therapy for these patients.

**Table 1:** Case series of Rmab therapy in CNSV and AAV. NG: Not given; DEI: Disease extent index; PVCNS: Primary vasculitis of the central nervous system; RTX: Rituximab; AAV: ANCA-associated vasculitis; CYC: Cyclophosphamide; MTX: Methotrexate; GC: Glucocorticoids; MMF: Mycophenolate mofetil; AZA: Azathioprine; CNSV: Central nervous system vascullitis.

Author-Year	Age (years); gender	Previous therapy	Rmab indication	Clinical finding and status at Rmab initiation	Imaging before Rmab	Concomitant therapy	Response after Rmab	Flares after Rmab	Follow-up in months (median)
Ruiz-Nieto N et al. (2023)[14]	62.1; 1F,6M	Initial treatment: high-dose corticosteroid therapy, followed by immunosuppressants.	A new-onset neurological or mental disability with an unclear cause after initial evaluation; PVCNS angiography or histology.	motor symptoms and speech alterations (n=2), headache (n=2), focal neurological signs (n=4)	Vascular lesions (n=4), inflammatory lesions (n=3)	Five patients received CYC, two received Rmab (one first-line and one second-line), and one received MTX.	Opportunistic infections and immunosuppressive therapy complications (4/7). Deaths: 4/7	Six instances progressed unfavorably, with four fatalities.	6-25 (23)
Krishna R et al. (2021) [15]	Rmab: age: 63 years; 10F, 1M; CYC: 69 years; 2F,4M	Previous diagnosis of vasculitis: In Rmab: maintenance immunosuppression: n=3; MTX: n=1; Rmab: n=1 In CYC: MTX: n=3, and AZA: n=2 for maintenance	Initial remission induction in patients who were never before diagnosed with AAV and suddenly had active disease, or remission induction on relapse in formerly remitted patients.	CNS involvement as the first manifestation: Rmab: 6/11 CYC: 1/6	MRI in Rmab: complete involvement: 5/11 Partial involvement: 4/11 None: 2/11 CYC: complete involvement in 100% of cases	Maintenance therapy: Rmab: 9/11 patients; 1/11 received MTX and 1/11 received AZA when MTX failed. CYC group: 2/6 AZA, 2/6 mycophenolate mofetil, and 2/6 renal transplant immunosuppression with cyclosporine or tacrolimus.	Initial induction remission in all CYC patients (100%) and 10 Rmab patients (91%). Two patients did not respond to Rmab for remission induction: one at diagnosis and one after recurrence. Death: 2 individuals per group	Rmab: 4/11; CYC: 1/6	9-127 (38)
Salvarani C et al. (2019) [16]	50.5 / 3F,3M	High- dose oral GCs, IV pulse methylprednisolone therapy, and conventional immunosuppressive agents	Refractory	headache, confusion (3 patients), and cognitive dysfunction (2 patients). One patient had symptoms related to spinal cord involvement, while one patient had Hodgkin's lymphoma, which occurred simultaneously.	MRI showed several bilateral infarcts in 2 patients and extensive leptomeningeal enhancement in 2 others, including the thoracic spine. On MRI, one patient exhibited a tumor-like mass with edema and mass effect, while another showed enhanced numerous cortical hemorrhagic regions.	Additional oral prednisone medication (median: 55 mg/day, range: 20-80 mg/day) was administered alongside Rmab. Two patients obtained Rmab in addition to MMF and AZA, whereas three got intravenous pulse methylprednisone one g/day for 3–5 days.	RTX improved clinical and MRI outcomes for all patients except one, who remained stable for three months following the second session.	18/3	16 – 105 (51)
Patel S et al. (2018) [17]	52 years; 1F/2M	Prednisolone 25 mg daily	refractory disease (n=2), induction therapy (n=1)	Sudden onset facial droop, slurred speech and confusion, sudden onset global aphasia (n=1)	Right internal capsule infarct, small infarcts involving the thalamus, prominent vessel attenuation, and irregularity involving the right middle cerebral artery and left posterior cerebral artery	Maintenance therapy: case 1: Two doses of i.v. Rmab (1 g), 10 mg prednisolone, 1mg CYC, 2g MMF Case2: three doses of 1 g i.v. Methylprednisone- lone, six cycles of 500 mg i.v. CYC and two doses of 1 g Rmab Case 3: three doses of 500 mg i.v. methyl- prednisolone followed by a weaning course of oral prednisolone and two doses of 1 g i.v. Rmab, and AZA (2mg/kg)	Three months after the second treatment of Rmab, a repeat MRI of the brain indicates a tiny new area of T2 hyperintensity but is generally stable. The patient remained clinically sound on prednisolone and oral CYC tapering.	Progressive cognitive impairment	6-9

**Table 2:** Non-randomized studies of Rmab therapy in CNSV or AAV. NG: Not given; DEI: Disease extent index; Rmab: Rituximab; AZA: Azathioprine; GC: Glucocorticoid; ANCA: Anti-neutrophil cytoplasmic antibody; AAV: ANCA-associated vasculitis; MTX: Methotrexate, PVCNS: Primary vasculitis of the central nervous system; PR3: Proteinase 3; EGPA: Eosinophilic granulomatosis with polyangiitis; GPA: Granulomatosis with polyangiitis; ELISA: Enzyme-linked immunosorbent assay; IQR: Interquartile Range; IV: Intravenous; ESRD: End-stage renal disease; CNSV: Central nervous system vasculitis.

Author-Year	Study design	Country	Mean age and gender	Disease assessment and premedication	Rmab dosage (induction or maintenance)	Concomitant therapy	Associated condition	Follow-up duration	Remission/relapses	Study Inference
Sorin B et al. (2022) [18]	Retrospective	France	45y (32–53) 10F,7M	NG	Rmab alone (375mg/m^2^/week regimen for four consecutive infusions) and MTX dose 25mg/week	Prednisone 40 mg/day,	Uncontrolled AAV (MPO and PR3)	11 months (11–26months)	Partial response in 11 (65%) and complete response in 4 (24%), failure 2 (12%) at 6m	Rabm/MTX combination treatment may be an appropriate treatment option for chronically active GPAs with granulomatous symptoms.
Agarwal A et al. (2022) [19]	Retrospective and prospective	India	34 (27.8–42) years, 14F,68M	Modified Rankin scale scores: 2 (0-3)	Treatment includes steroids only, steroids along with CYC (750 mg/m^2^), AZA (2–3 mg/kg/day), MTX (15–25 mg/week), MMF, or Rmab (375 mg/m^2^ iv infusion/week for four weeks followed by an additional infusion at six months, if indicated).	NG	PVCNS confirmed by brain biopsy or cerebral digital subtraction angiography	2 (0-3y)	65.2% had good functional outcomes.	Taking steroids and additional immunosuppressants possibly lowered relapse rates.
Thietart S et al. (2022) [20]	Prospective	France	79.4 (76.7-83.1) 51F, 42M	BVAS: 14 (10-19)	375 mg/m^2^ iv infusion/week for four weeks or two 1 g Rmab infusions 15 days interval	High-dose GC to start at 1mg/kg. Nine patients had CYC, and 10, 2, and 1 got plasma exchange, MTX, and immunoglobulin.	Induction and maintenance regimens for ANCA (GPA and MPA)	2.3 (1-1.4)	As induction therapy, 57/66 patients remitted. Death rate: 19.7/100-patient-years (induction therapy), 5.3/100-patient-years (maintenance).	Maintenance with Rmab reduced relapse and severe infections. Remission with severe infection increased with the induction regimen.
Gulati K et al. (2021) [21]	Retrospective	UK	66 (22 – 84), 25F, 39M	BVAS: 19 (16-23)	Rituximab (2g), low-dose intravenous CYC (10mg/kg), oral prednisolone (1mg/kg/day), and plasma exchange (60 ml/kg)	Azathioprine (29/64), and with MMF (4/64) or Rmab (18/64)	Induction and maintenance regimens for ANCA (MPO and PR3)	46 (25-65) months.	Overall, patient survival was 85%, and 69% were ESRD-free.	In severe AAV, combined immunosuppressive therapy may allow GC abstinence and quick and long-term disease management.
Mittal S et al. (2021) [22]	Retrospective	India	40 (IQR 29–53)y 49F/28M	BVAS at induction: primary induction - 19 (IQR 11.75–22.5); reinduction at relapse - 6 (IQR 5–16.5)	Intravenous 1 g Rmab, two infusions 15 days apart	After three days of IV methylprednisolone pulse (1 g/day) oral prednisolone (1 mg/kg/day) for four weeks. Reinduction upon relapse: MTX, AZA, CYC	GPA remission induction or maintenance therapy	27 (IQR 10–40)m	Relapse at 6m: 8/25 (primary induction), 6/35 (reinduction at relapse)	Rmab benefited GPA patients to achieve and maintain remission.
Smith RM et al. (2020) [23]	Prospective	UK	59 (19-89), 93F/95M	BVAS: 5 (baseline), 149/188 had previously received CYC, and 67/188 Rmab.	Therapy to induce remission 4×375 mg/m^2^ Rmab maintenance phase: 1000 mg Rmab at four monthly fixed intervals or 2 mg/kg/day AZA	High or low-dose GC regimen: 1 mg/kg/day or 0.5 mg/kg/day to 10 mg/day in four months	Relapsing GPA or MPA	12-24m	171/188 (90%) patients remitted within four months. Only six (3.2%) individuals had uncontrolled illness at four months. Four patients died during induction.	The trial showed high efficacy for reinducing remission in relapsing AAV patients.
Pepper RJ et al. (2019) [24]	Prospective	UK	Group1: 65 (16-85y) 9F/14M Group2: 66 (43-83) 16F/10M	BVAS (baseline): Group1: 17 (12-29); Group2: 16 (7-24)	Group1:Rmab 2x1g (0 and 7 days), CYC 6x500-750 mg pulses 0, 2, 4, 6, 8, 10 Group 2: Rmab 2x1g (0 and 14days), CYC 6x500 mg pulses 0, 2, 4, 6, 8, 10 Maintenance: AZA 2mg/kg	Group 1 received oral prednisolone (2x 250-500 mg) for seven days and group 2 for 14.	New diagnosis or relapsing AAV.	24m	Three deaths and two relapses at months 7, 9, and 12.	Early GC cessation in severe AAV induces remission as well as the usual course of therapy and reduces GC-related side effects.
Teixeira V et al. (2019) [25]	Retrospective	UK	51 (39.5–58y) 44F/25M	BVAS: 6 (baseline), 1 (6-months), 0 (1y, 2y). Hydrocortisone 100 mg iv, chlorpheniramine 10 mg iv or diphenhydramine 50 mg and oral acetaminophen 1 g before each Rmab infusion; and PCP with sulfamethoxazole/trimethoprim for six months	Rmab two doses of 1000 mg 2 weeks apart, with only five patients receiving a dose of 375 mg/m2/week for four weeks.	Prednisolone dose (mg/day, median) decreased from 12.5 to 7, 7.5, and 5 at 6, 12 and 24 months, respectively	Refractory EGPA	12.25-24m	54% relapsed by 24 months. Rmab had an 84.8% 12-month survival rate and a 68.4% 24-month rate.	Although Rmab reduced prednisolone use and improved EGPA, asthma, and ENT relapse rates remained high.
Puéchal X et al. (2019) [26]	Prospective	France	45 (IQR 30-59) 64F, 50M	BVAS: 9 (6-14). Methylprednisolone (100 mg), paracetamol (1 g) and dexchlorpheniramine (5 mg), PCP prophylaxis	Four 375mg/m^2^ Rmab infusions/week or two 1g infusions two weeks intervals. All patients receive 500 mg Rmab every six months until 24 months.	Methylprednisolone, one g/day for 1-3 days, before oral prednisone, if necessary by the treating physician, and oral prednisone up to 1 mg/kg/day.	Induction and maintenance therapy for refractory or relapsing GPA	3.6 (1.6-5.8) years	85% 2-year relapse-free survival and 78% Rmab retention rates	Inducing GPA remission with Rmab and low-dose preventive maintenance was secure and effective
Roccatello D et al. (2017) [27]	Prospective	Italy	5 GPA, 4 MPA, 2 EGPA	BVAS: 0 at the end of follow-up.	On days 1, 8, 15, and 22, 375 mg/m^2^ Rmab was injected systemically. An additional two were given one and two months following the last dose.	Oral prednisone was tapered to 5 mg/day by the end of the third month after Rmab.	Refractory AAV	45-132 (85) months	Seven relapsed patients received Rmab at a median of 54 months.	A 4+2 Rmab infusion strategy was used for AAV resistant to standard treatment for prolonged immunosuppressive-free clinical remission.
Lionaki S et al. (2017) [28]	Retrospective	Greece	Rmab group: 51; 9F, 5M Control group: 47; 11F/10M	BVAS (after therapy): 10.5 (Rmab); 12 (control)paracetamol, bilusepin, and hydro cor- this one were used in each rmab ministration, and a course of sodium 2-sulfanylethanesulfonate before each CYC pulse	Rmab group: 375 mg/m^2^ for four weeks or 1 gram every two weeks, twice, along with CYC+steroids. Control group: CYC was given iv (500-1000 mg/m^2^) or orally (1.5-2.0 mg/day/kg) with GC (3 g methyl-prednisolone pulses, accompanied by oral weaning or prednisone commencing from 1 mg/kg/day).	Maintenance treatment: AZA, MMF, or MTX	Multiple-relapsing AAV	Rmab group: 30.5 (5–68) Control group: 36 (3–228)	35 (23.8%) had at least one major relapse	After a significant relapse, successive therapy with CYC and Rmab in frequently relapsing AAV patients prolongs remission, reducing exposure to the drug.
McGregor JG et al. (2015) [29]	Retrospective	USA	Rmab: 50y (75%F) CYC: 60y (46%F) Rmab+CYC: 50y (49%)	Diagnostic biopsy and a positive ANCA determination by immunofluorescence, ELISA	Rmab: 375 mg/m^2^/week for four doses CYC: 1 IV dose or one month of oral CYC Rmab+CYC:	Prednisone, CYC, MMF, and AZA	Refractory and maintenance AAV	3-48m	Complete remission: n=21	Rmab and CYC administered during active illness before remission may prolong recovery longer than Rmab alone.
Timlin H et al. (2015) [30]	Retrospective	USA	71±6, 21F/10M	BVAS/WG: 4.4 ± 1.5 Prednisone	Rmab 375 mg/m2, and one patient received 1000 mg every two weeks for two doses.	Oral CYC for median of 52 days. GC was utilized in 29 of 31 individuals. Every patient received PCP prophylaxis.	Remission induction of GPA or MPA	1061±972 days	Thirty patients attained remission, with a mean period of 57±27 days. The remission maintenance regimen comprised Rmab, AZA, and MFF.	Rmab induces remission in geriatric AAV patients. Also, infectious problems were common.
Chocova Z et al. (2015) [31]	Retrospective	Czech Republic	37.5y, 7F/11M	BVAS, paracetamol, bisulepin and hydrocortisone was used in each Rmab session	In 12 individuals, Rmab was given in two CYC pulses at two two-week intervals. In six individuals, only GC were utilized.	As induction treatment, four patients received 375 mg/m^2^ of BSA every seven days for four weeks, and 14 patients had two 1 g injections after two weeks. Maintenance pre-emptive therapy was 1 g every six months for two years with Rmab.	Refractory AAV	3-82 (25.5m)	Five patients (33.3%) had partial remission at six months, and eight (53.3%) complete remission. Three infected patients died.	For most AAV patients, Rmab reduced GC and eliminated cytotoxic medications.
Alberici F et al. (2015) [32]	Retrospective	UK	52.02y, 41F, 28M (GPA 62/69, MPA 7/69)	DEI=2.5; premedication with i.v. Hydrocortisone 100mg, i.v. Chlorpheniramine 10mg and oral paracetamol 1g before the infusion.	Induction therapy: 375mg/m^2^/week for four weeks; maintenance therapy: 1 g every six months for 24 months.	Oral prednisolone at 10mg/day, six i.v. CYC, two plasma exchanges, 1 AZA, and 1 MMF.	Refractory AAV	59.3m	Relapses 28/69 (median 15.5m). Two individuals died, and two had severe hypogammaglobulinemia.	A fixed-interval Rmab maintenance strategy efficiently minimizes relapse in refractory AAV.

**Table 3:** Randomized controlled studies of Rmab therapy in CNSV and AAV. AAV: Anti-Neutrophil Cytoplasmic Antibody-Associated Vasculitis; CNSV; Central Nervous System Vasculitis; AZA: Azathioprine; GC: Glucocorticosteroids; Rmab: Rituximab; CYC: cyclophosphamide; MTX: methotrexate; NG: Not given; VDI: Vasculitis Damage Index; BAS: Birmingham Vasculitis Activity Score; ESRD: End-stage renal disease; ANCA: Anti-neutrophil cytoplasmic antibody; GPA: Granulomatosis with polyangiitis; MPA: Microscopic polyangiitis.

Author-Year	Study design	Country	Mean age	Sample size and gender	Therapeutic regimen	Outcome measures and follow-up	Study Inference
Smith RM et al. (2023) [33]	International randomized controlled, open-label, superiority trial (RITAZAREM)	29 centres in seven countries	Rmab: 57.1±15.1 AZA: 58.6±13.9	Rmab: 42F/43M; AZA: 44F/41M	Either AZA (2 mg/kg/day, reduced after month 24) or Rmab (1000 mg iv every four months, through month 20)	Serious adverse events: Rmab: 19/85; Death: n=3 AZA: 31/85; Death: n=1	For patients with AAV with a history of relapse, fixed-interval, repeat-dose Rmab was more effective than AZA in averting recurrence of the disease after Rmab was used to induce remission for at least 36 months.
Furuta S et al. (2021) [34]	Phase 4, multicenter, open-label, randomized, noninferiority trial (LoVAS).	Japan	Low-dose GC+Rmab: 66-78 High-dose GC+Rmab: 68-78	Low-dose GC+Rmab: 43F/26M High-dose GC+Rmab: 37F/28M (AAV)	Low-dose GC Prednisolone 0.5 mg/Kg/d, High-dose GC Prednisolone 1 mg/Kg/d, and Rmab 375 mg/m^2^/week	Death (2 in low-dose and 3 in high-dose groups), relapse (3 in low-dose and 0 in high-dose groups), ESKD (0 in low-dose and 1 in high-dose groups), and remission rate (after six months, 49 in low-dose and 45 in high-dose groups)	A reduced-dose GC plus Rmab regimen was non-inferior to a high-dose GC plus Rmab regimen, inducing a disease-free state at six months in patients with recently diagnosed AAV who did not have severe glomerulonephritis or alveolar hemorrhage.
Charles P et al. (2020) [35]	Multicenter, double-blind RCT (MAINRITSAN3)	39 clinical centers in France	Rmab: 64.6±10.7 Placebo: 63.1±11.2	Rmab: 15F/35M (32 GPA, 18 MPA) Placebo: 19F/28M (36 GPA, 11 MPA)	Methylprednisolone iv (100 mg), dexchlorpheniramine (5 mg), and acetaminophen (1000 mg) before Rmab and placebo infusions. Infusions of 500 mg Rmab biannually for 18 months and placebo	Relapse-free survival at month 28 (Rmab: 96% and placebo: 74%), relapse (Rmab: 2), BVAS, VDI at 28 months (Rmab: 2.2±1.8; placebo: 1.7±1.6)	Prolonged therapy with biennial Rmab infusions over 18 months reduced AAV recurrence as opposed to maintenance therapy.
Charles P et al. (2018) [36]	Open-label, pragmatic, multicentre, RCT (MAINRITSAN2)	39 clinical centers in France	Fixed schedule: 59±13 Individually-tailored: 62±14	Fixed schedule: 37F/44M (61 GPA, 20 MPA) Individually-tailored: 31F/50M (56 GPA, 25 MPA)	All instances require intravenous methylprednisolone (100 mg), dexchlorpheniramine (5 mg), and acetaminophen (1000 mg). Regulated schedule: 500 mg Rmab infusion on days 0 and 14 post-randomization, then 6, 12, and 18 months later. Individually tailored: 500 mg Rmab infusion at randomization, reinfusion only when Cd19+B cells or ANCA recurred, or ANCA titer increased significantly.	There were 83.8% relapse-free survival rates in tailored and 86.4% in a fixed schedule, with BVAS and VDI (1.99±1.57 for tailored and 2.09±1.97 for the fixed schedule at 28 months).	Relapse rates were similar for individually designed and fixed-schedule Rmab courses.
Terrier B et al. (2018) [37]	Prospective, open-label RCT (MAINRITSAN)	France	NG	Rmab (n=57), Azathioprine (n=58)	Rmab (500 mg on days 0 and 14, and at months 6, 12, and 18 postinclusion) infusions and AZA (2 mg/kg/day for 12 months; 1.5 mg/kg/day for six months; 1 mg/kg/day for four months) for remission maintenance	Major relapses occurred between 28-60 months with Rmab (n=13) and AZA (n=11). Minor relapses: Rmab (n=7), AZA (n=3). Relapse-free survival rates at 60 months: AZA 49.4%, Rmab 71.9%. Overall survival: 93% AZA, 100% Rmab.	Rmab-based maintenance regimens improved overall survival and remission for AAV patients over 60 months.
Pugnet G et al. (2016) [38]	Phase III RCT (MAINRITSAN)	France	AZA: 56±14 Rmab: 54±13	50F/65M (87 GPA, 23 MPA, renal limited cases)	AZA maintenance therapy at 2 mg/kg/d for 22 months or Rmab 500 mg infusion on days 1 and 15, 5.5 months after that, and every six months for five infusions over 18 months.	Health Assessment Questionnaire scores: Rmab: 0.24±0.38 AZA: 0.33±0.53. Short-form Health Survey (SF-36) physical component score was better for Rmab (+3.95 points, p=0.067), while the mental component score was better for AZA (+4.23 points, p=0.041).	AZA-treated AAV maintenance patients had a deterioration in physical capacities at 24 months as opposed to Rmab.
Jones RB et al. (2015) [39]	Two parallel limbs, RCT (RITUXVAS)	UK	NG	Rmab: (n=33); CYC/AZA control group (n=11)	Patients received GC and either Rmab (375 mg/m2/week×4) with two iv CYC pulses (n=33, Rmab group) or CYC for 3–6 months followed by AZA (n=11, control group)	Death, ESKD, and recurrence in Rmab: 14/33; control group: 4/11	At 24 months, ESRD, mortality, and relapse rates were similar between groups. Relapse was linked to B cell reappearance in the Rmab group.
Geetha D et al. (2015) [40]	Multicenter, double-blind, double-dummy, placebo-controlled RCT (RAVE)	USA	Rmab: 53F/47M CYC/AZA: 43F/57M	Rmab: 56±15; CYC/AZA: 54±13 (GPA: MPA: 31)	Premedication comprises one to three 1000 mg methylprednisolone pulses and 1 mg/kg/day prednisone dosages (maximum 80 mg/day). Rmab (375 mg/m^2^/week for four weeks with high-dose GC lowered to 0 mg/day over six months and no maintenance therapy). Control group: CYC for 3-6 months after that AZA for 18 months after remission.	Complete remission at 18 months of treatment: Rmab: n=38; CYC/AZA: n=39 (*P*=0.82).	Rmab and CYC induce remission in AVA and the renal system equally.

The case series suggests that Rmab is a good replacement therapy for PVCNS patients who do not respond to conventional therapies or have contraindications [17]. Rmab also caused prolonged remission in PVCNS patients, with follow-up periods of 51 months (IQR: 16-105) in one trial and 38 months (IQR: 9-127) in another [15].

Clinically diverse patients were studied for Rmab efficacy in non-randomized studies. According to several studies, Rmab induces remission in refractory AAV, EGPA, uncontrolled and relapsed AAV, and in both induction and maintenance therapy [20-22, 26]. Thietart S et al. [20] evaluated Rmab for induction and maintenance in AAV individuals over 75. Induction treatment with Rmab led to remission in 86.4% of patients, while maintenance therapy had a reduced relapse rate. Another investigation found that Rmab induces remission in 96.7% of AAV patients aged 60 and above but has an elevated infection rate [30]. Due to a greater possibility of therapy-related adverse events in AAV individuals under CYC or GC therapy, Rmab appears intriguing for older people [30]. Rmab infusion amounts (4×375 mg/m2 and 2×1 g) did not affect therapeutic response or relapse [29].

Roccatello D et al. [27] found that six doses of 375 mg/m2 Rmab were successful in sustaining remission without maintenance treatment and can be recommended for cases refractory to immunosuppressive medication. In 18 AAV patients, Chocova Z et al. [31] found that 72% achieved remission (complete or partial) with Rmab therapy, whereas those with lung, ENT, or renal involvement did not.

The RITUXVAS trial researched the long-term effects of Rmab on AAV individuals with renal disease. At 24 months, Rmab and CYC had similar mortality rates, end-stage renal disease (ESRD), and relapse rates [39]. A RAVE trial subgroup study of 197 AAV individuals with renal failure confirmed this perspective, showing that four doses of 375 mg/m2/week of Rmab with GCs contrasted with 18 months of sequential treatment with 2 mg/kg/day CYC, which was substituted by 2 mg/kg/day azathioprine (AZA) 3 to 6 months later, resulted in no significant differences in renal function outcomes [40]. The MAINRITSAN studies revealed improved quality of life for AAV patients in the Rmab group compared to the AZA group. After 60 months of MAINRITSAN study follow-up, Rmab patients had more sustained remission than AZA patients (71.9% vs. 49.4%) [37]. A phase 3 clinical trial compared the conventional and specific Rmab infusion methods. In MAINRITSAN2, 162 adult AAV individuals in full remission participated. Every patient received 500 mg Rmab; however, the traditional group received it on days 0 and 14 after randomization, followed by months 6, 12, and 18. The second group was given Rmab at randomization and re-administered when B-lymphocytes or ANCA titers elevated sufficiently. The personalized Rmab infusion and the predetermined infusion had similar relapse rates in the MAINRITSAN2 study [36].

The MAINRITSAN3 trial involved 97 AAV cases in complete remission from the MAINRITSAN2 investigation (median age: 64.6±10.7 years) with no severe relapse. For maintenance, individuals were assigned to placebo or Rmab. MAINRITSAN3 was conducted to extend the follow-up from MAINRITSAN2 to determine the appropriate Rmab maintenance therapy duration. Four doses of 500 mg Rmab were injected every six months for 18 months before being compared to a placebo after 28 months. Disease relapse was reduced in the Rmab group compared to the placebo [35]. The LoVAS clinical trial examined the efficacy of prednisolone in Rmab-treated AAV individuals at 0.5 and 1 mg/kg/day. At six months, 71% and 69.2% of the low- and high-dose GC subjects achieved remission, respectively, revealing statistically insignificant differences [34].

Prospective research comprised 49 ANCA-positive or renal-limited vasculitis cases. The patients received low-dose CYC, Rmab, and 7- or 14-day GC. A short-term or long-term GC strategy for remission induction had similar results, but GC-associated side events were reduced [24]. A retrospective French national investigation of 17 persistent AAV patients who failed Rmab or CYC demonstrated that Rmab and MTX combination treatment was highly effective in persistently active GPA with significant granulomatous presentation [18]. The addition of CYC to Rmab increased suppressed immune agents, promptly improving renal inflammation and function [21]. Another investigation found that Rmab with CYC among severe relapse cases offers prolonged remission and reduces CYC usage [28].

GC and one other immunosuppressive medication were typically provided in most studies [18-24,28-32,39,40. The most important supplementary medication is CYC. Other induction therapies include IV immunoglobulins, infliximab, plasma exchange, GC, AZA, MMF, Rmab, or MTX. Often, immunosuppressants and oral GC are tapered for maintenance therapy. In 5 of 30 studies, Rmab was utilized for induction (2x1000 mg infusions in two-week intervals or 375 mg/m^2^/week for four weeks) and maintenance (1,000 mg/month for six months). Rmab improved neurologic signs and imaging and reduced relapses in patients refractory to standard immunosuppressive treatments [16,17].

Discussion

Rmab therapy can induce remission in patients with active disease, but most will experience relapse within the first year due to CD20+ B-lymphocyte regeneration. To prevent disease recurrence, preemptive maintenance therapy is often used, although there is ongoing debate about its necessity in asymptomatic patients and the optimal dose, timing, and safety of such regimens. Studies have shown that Rmab is effective and safe in managing chronic relapsing cases, with a significant increase in relapse-free survival regardless of maintenance dose or interval. Management strategies may involve a fixed schedule (e.g., every six months) or adjustments based on B-cell repopulation and ANCA titers [10]. To distinguish between diverse research outcomes, Ma TT et al. [41] specified three features related to management insights using the Birmingham Vasculitis Activity Score (BVAS). This score characterized complete remission as a complete lack of disease activity requiring sustained immunosuppressive medication, and partial remission as a 50% drop in disease activity score without introducing new symptoms. Nevertheless, relapse was characterized as the recurrence of new disease symptoms suggesting inflammation after the patient had achieved remission.

Salvarani C et al. [42] noted that small and medium vessels, including leptomeningeal and parenchymal arteries, are primarily affected, resulting in vasculitis affecting medium vessels in 45 to 69% of patients and small vessels in 23 to 30%. Involvement of small and medium vessels is a frequent hallmark of primary central nervous system vasculitis (PVCNS) and ANCA-AAV. PVCNS can present with a variety of clinical symptoms. However, Nehme A et al. [43] stated that it can occasionally be impractical to diagnose primary vasculitis without additional workup, including CNS biopsy, radiological features, and a comprehensive clinical evaluation. Physicians face great challenges in diagnosing and treating both primary and secondary vasculitis because there is no conventional presentation, and both illnesses include symptom mimickers [43]. In addition, the similarities in neurological symptoms between the two illnesses, as well as the dearth of standardized radiological results specific to vasculitis, are reflected in the lack of an optimal diagnostic workup for prompt diagnosis [44].

To combat this issue, the MAINRITSAN2 enrolled GPA and MPA patients. As a result, as maintenance treatment, certain individuals received 500 mg Rmab infusion if their CD19+ B-lymphocytes had repopulated or if their ANCA had increased until 18 months; controls received a fixed 500 mg dose of Rmab every six months, regardless of their cell population or ANCA titer. At 28 months, most patients in both groups claimed prolonged remission, but 17.3% in the conditional Rmab group and 9.9% in fixed-schedule cases experienced illness relapse, which was statistically insignificant. Interestingly, the conditional Rmab group participants experienced fewer infusions than the control [36].

A post-hoc trial of the MAINRITSAN2 study was performed to assess the efficacy of different numbers of initial Rmab infusions. This research demonstrated insignificant differences in the prolonged remission rate over 12 months, comparing those who received Rmab on day one and day 14 against those who had a single 500 mg infusion as an induction, implying that the second infusion was unnecessary [45]. Takeyama Y et al. revealed no substantial distinction between Rmab maintenance treatment and traditional immunosuppressive drugs. Nevertheless, the cases improved with lower doses of GC [46]. Most investigations on preemptive Rmab maintenance therapy safety found no significant concerns [33,34,37,40]. In contrast, Besada E et al. [47] documented that despite reducing the probability of relapse, around 33% of patients discontinued Rmab after a median follow-up of 41 months due to adverse effects, primarily hypogammaglobulinemia and infections. However, no studies reported a disease relapse within a median of 14.5 months.

The possible adverse effects of Rmab, such as infection, malignancy, hypersensitivity, cytopenia, and hypogammaglobulinemia, should be discussed with a medical professional [10]. To avoid or reduce such adverse effects, it is urged to screen for chronic infection before Rmab therapy and to use a preemptive regimen, such as vaccination against bacterial and viral agents, keeping track of white cell count and immunoglobulin levels, and prophylaxis against frequently reported ailments [10,48,49]. Along with safety-related issues, some deaths were reported following Rmab infusion, which could be attributed to infection, cardiovascular difficulties, neurological effects, malignancy, or respiratory failure [10]. It has also been demonstrated that the risk of death associated with Rmab therapy is greater when Rmab is administered to induce remission rather than for maintenance [20]. During Rmab treatment, healthcare providers should evaluate and manage the potential adverse effects prudently to avoid fatality.

In the therapeutic context, we recommend employing Rmab as the initial therapy option, particularly in those cases where other standard immunosuppressive drugs are contraindicated. Further investigations into the appraisal of combination therapy and long-term patient follow-up to evaluate safety might shed light on additional facets of Rmab. As the therapeutic procedures, clinical, and radiological presentations of PVCNS and AAV with CNS involvement exhibit similarities between the two conditions; future studies should focus on developing a viable diagnosis technique.

## Conclusions

Rmab has emerged as an effective option for treating secondary CNS vasculitis and AAV, helping to achieve remission and reduce relapses, especially in patients who do not respond well to conventional therapies. However, while it is beneficial in these conditions, its use in PVCNS remains less clear. The potential benefits of Rmab, such as preventing relapses, need to be carefully weighed against risks like infections, which require close monitoring. More research is needed to understand its role in PVCNS, to develop targeted treatment strategies, and to improve diagnostic tools to distinguish between different types of CNS vasculitis.
